# Metagenome-assembled genome sequence of *Candidatus* Electrothrix sp. NPCB-01 from Southern California marine sediments

**DOI:** 10.1128/mra.00025-26

**Published:** 2026-03-06

**Authors:** Sukrampal Yadav, Tingting Yang, Magdalene A. MacLean, Mohamed Y. El-Naggar

**Affiliations:** 1 Department of Physics and Astronomy, University of Southern California 5116, Los Angeles, California, USA; 2 Department of Biological Sciences, University of Southern California 5116, Los Angeles, California, USA; 3 Department of Chemistry, University of Southern California 5116, Los Angeles, California, USA; Montana State University, Bozeman, Montana, USA

**Keywords:** cable bacteria, extracellular electron transport, metagenome, nickel-dependent long-distance electron transport

## Abstract

Cable bacteria conduct long-distance electron transport in sediments but are not yet isolated in pure culture. We report the metagenome-assembled genome of *Candidatus* Electrothrix sp. NPCB-01 from Newport Bay, California. This 3.46-Mb genome encodes sulfur oxidation, nitrogen and carbon metabolism, and nickel homeostasis genes, expanding resources for these electroactive microbes.

## ANNOUNCEMENT

Cable bacteria are filamentous, multicellular microbes capable of conducting electrons over centimeter-scale distances, coupling sulfide oxidation in anoxic sediments to oxygen reduction near the surface (1–4). This long-distance electron transport (LDET) is mediated by periplasmic conductive nanofibers recently proposed to contain nickel cofactors (5–7). The clade comprises two described genera occurring globally in diverse sedimentary environments: *Candidatus* Electrothrix (marine) and *Candidatus* Electronema (freshwater) (8–11). Here, we report the metagenome-assembled genome (MAG) of *Candidatus* Electrothrix sp. NPCB-01, which was not isolated in pure culture but reconstructed from metagenomic sequencing of marine sediments collected from the Upper Newport Bay State Marine Conservation Area (33° 38′52″ N, 117° 52′21″ W), Newport, California.

Total DNA was extracted from 1-month incubated sub-surface sediments (*n* = 2) using the DNeasy PowerSoil Pro Kit (Qiagen, Germany). DNA quantity and integrity were assessed using a Qubit fluorimeter and agarose gel electrophoresis. Paired-end libraries were prepared using the NEBNext Ultra DNA Library Prep Kit and quantified using an Agilent TapeStation High-Sensitivity D1000 ScreenTape. Shotgun metagenome sequencing was performed on an Illumina NovaSeq (2 × 250 bp 50 million) at MR DNA Lab (Shallowater, TX, USA), yielding 129.7 and 210.9 million reads.

Reads were merged into a single library using kb_ReadsUtilities (v1.2.2) (12), quality-checked with FastQC (v0.12.1) (13), and trimmed using Trimmomatic (v0.39) (14). Contigs were assembled with MEGAHIT (v1.2.9) using the meta-sensitive preset (15). Genome bins were generated using CONCOCT (v1.1.0) (16), MetaBAT2 (v1.7.0) (17), and MaxBin2 (v2.2.4) (18), assessed with CheckM (v1.0.18) (19) and refined with MetagenomeUtils (v1.1.1) (12). High-quality bins were annotated in RASTtk (v1.073) (20), taxonomically classified with GTDB-Tk (v2.3.2) (21), and placed phylogenetically using SpeciesTree analysis (v2.2.0) (22). To predict metabolic potential, genome-scale metabolic models were constructed using the ModelSEED-based Build Metabolic Model App (fba_tools v2.2.1) (23, 24). Default parameters were used unless otherwise noted.

**Fig 1:**
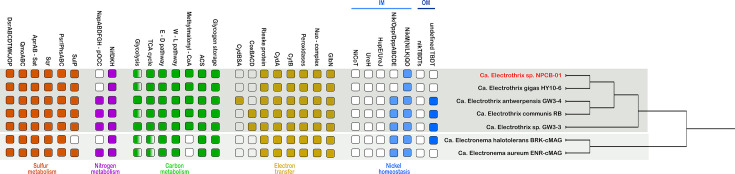
Comparative metabolic potential and phylogenetic placement of *Ca*. Electrothrix sp. NPCB-01. The heatmap displays the presence (colored squares), absence (empty squares), and partial presence (gradient squares) of key metabolic genes across related *Candidatus* Electrothrix and *Candidatus* Electronema genomes. The phylogenetic dendrogram (right) illustrates the taxonomic placement of the NPCB-01 MAG (red) within the *Ca*. Electrothrix genus.

The *Ca*. Electrothrix sp. NPCB-01 MAG (3,457,740 bp; 46.1% G+C content) comprises 104 contigs, 3,566 coding sequences, and 43 RNAs (Table 1). CheckM analysis with lineage-specific marker sets indicates the genome is ~95.8% complete. A phylogenetic dendrogram places NPCB-01 within the *Ca*. Electrothrix genus, confirming its affiliation with marine cable bacteria, while a comparative heatmap highlights its distinct metabolic features (Fig. 1). The genome encodes a near-complete sulfur metabolism module, including dissimilatory sulfite reductase (*dsrABCD*) and adenylylsulfate reductase (*aprAB-sat*), crucial in the sediment sulfur cycle. It encodes mostly complete carbon metabolism pathways, though it lacks a complete glycolysis pathway. Furthermore, the genome contains key electron transfer components and a full suite of nickel homeostasis genes (*nikM*/*N*/*L*/*K*/*QDE* and *rcnA*), along with various nickel-binding carbon monoxide dehydrogenase (CODH) maturation genes (*cooC/F/J/S/T*) and genes encoding FNOR and CODH/ACS (*α*, *β*, *γ*, and *δ* subunits). These features are consistent with a chemoautotrophic and electroactive lifestyle that may be driven by a nickel-dependent LDET process, as previously proposed (11, 25–27).

**TABLE 1:** General features of the metagenome-assembled genome of *Ca*. Electrothrix sp. NPCB-01

Items	Description
Project	MAG of cable bacterium from Southern California
Sequencing platform	Illumina NovaSeq 6000
Assembly method	MEGAHIT
Genome coverage	98.5×
BioProject	PRJNA1334316
Accession number	GCA_053813925
Size (bp)	3,457,740
DNA G + C content (%)	46.13
CDSs	3,566
N50 value	57,709
L50 value	19
RNAs	43

*Ca*. Electrothrix sp. NPCB-01 MAG expands the genomic representation of cable bacteria from the Pacific coast of Southern California and offers a resource for studying the genetic, biochemical, and bioenergetic basis of electron transport in sediment ecosystems.

## Data Availability

The metagenome-assembled genome sequence of *Candidatus* Electrothrix sp. NPCB-01 has been deposited in the NCBI under PRJNA1334316 BioProject with GenBank assembly GCA_053813925 (ASM5381392v1). Raw sequencing reads are available in the Sequence Read Archive (SRA) under accession numbers SRR35613409 and SRR35613410.
